# Current Advances in Optimal Medical Therapy for Heart Failure

**DOI:** 10.3390/jcm14051417

**Published:** 2025-02-20

**Authors:** Teruhiko Imamura

**Affiliations:** Second Department of Internal Medicine, University of Toyama, 2630 Sugitani Toyama, Toyama 930-0194, Japan; te.imamu@gmail.com; Tel.: +81-76-434-2281; Fax: +81-76-434-5026

I am delighted to present this Special Issue, which focuses on the latest advancements in the optimal medical therapy for heart failure. This collection features five publications exploring key aspects such as optimal patient selection, risk stratification, and detailed management strategies leveraging recently available heart failure therapies.

Recent innovations in heart failure medications have significantly improved both mortality and morbidity outcomes for patients with chronic heart failure [1,2,3]. The clinical evidence supporting guideline-directed medical therapy—particularly with respect to the “Fantastic Four”, comprising angiotensin receptor neprilysin inhibitors, mineralocorticoid receptor antagonists, beta-blockers, and sodium-glucose cotransporter 2 (SGLT2) inhibitors—continues to grow [4,5].

Among these, SGLT2 inhibitors exhibit several distinctive features [6,7]. First, they are indicated for heart failure patients across a wide spectrum of left-ventricular ejection fractions [8]. Second, SGLT2 inhibitors have demonstrated efficacy in reducing mortality and morbidity irrespective of affliction with diabetes mellitus, although the precise underlying therapeutic mechanisms remain unclear.

SGLT2 inhibitors are also notable for their hypothesized adverse effects, such as the potential exacerbation of sarcopenia [9]. Heart failure is often complicated by sarcopenia, which substantially increases the risk of functional decline, hospitalization, and mortality. As glucose-lowering agents, SGLT2 inhibitors reduce hyperglycemia by inhibiting SGLT2 in the proximal renal tubule, leading to calorie loss via glucosuria. This calorie loss may, in turn, contribute to reducing lean body mass, including fat and muscle. Consequently, clinicians often hesitate to prescribe SGLT2 inhibitors to elderly patients due to concerns about aggravating sarcopenia. However, in a study by Horiuchi and colleagues [10], SGLT2 inhibitor therapy was associated with a lower incidence of frailty-related events. While further research is needed to establish robust evidence, these findings suggest that clinicians may not need to overly restrict SGLT2 inhibitor use in older populations.

Ivabradine, another recently introduced heart failure therapy [11], lowers heart rate by inhibiting the “funny current” in the sinoatrial node and has been shown to improve mortality and morbidity among patients with systolic heart failure and sinus tachycardia [12]. A current challenge is determining the optimal target heart rate during ivabradine therapy for individual patients [13]. Recent studies recommend using doppler transmitral echocardiography, wherein the optimal heart rate is achieved when the E-wave and A-wave are adjacent but not overlapping [14]. Yamamoto and colleagues advanced this concept by examining the PR interval, demonstrating that ivabradine prolongs this interval—a novel surrogate marker for favorable clinical outcomes among systolic heart failure patients [15]. Further investigation is needed to elucidate the clinical implications of PR-interval-guided ivabradine therapy.

Hypoxia-inducible factor-prolyl hydroxylase (HIF-PH) inhibitors have emerged as a treatment for renal anemia [16]. Given the complex cardio–renal–anemia interplay, aggressive management of renal anemia may improve outcomes for heart failure patients [17,18,19]. However, there is still no standardized approach for treating renal anemia in this population, particularly due to the adverse outcomes associated with erythropoietin-stimulating agents. Sezai and colleagues reported comparable clinical parameters following the transition from continuous erythropoietin receptor activators to HIF-PH inhibitors in patients with heart failure and renal anemia [20]. Further research is required to establish target hemoglobin levels during HIF-PH inhibitor therapy and clarify their impact on heart-failure-related parameters.

The studies presented in this Special Issue underscore the transformative potential of contemporary heart failure therapies, providing valuable insights into their clinical applications and mechanisms of action. By continuing to address unresolved questions and refining therapeutic strategies, I sincerely believe that we can further enhance the quality of care for patients with heart failure. I hope that this Special Issue inspires future research and contributes to the ongoing evolution of heart failure management.

## Data Availability

This manuscript does not include any original data.
